# Interest of low-dose hydrocortisone therapy during brain-dead organ donor resuscitation: the CORTICOME study

**DOI:** 10.1186/cc13997

**Published:** 2014-07-23

**Authors:** Michel Pinsard, Stéphanie Ragot, Paul Michel Mertes, Jean Paul Bleichner, Samira Zitouni, Fabrice Cook, Marc Pierrot, Laurent Dube, Edgard Menguy, Laurent Martin Lefèvre, Laurence Escaravage, Pierre-François Dequin, Philippe Vignon, Nicolas Pichon

**Affiliations:** Intensive Care Unit, Inserm U 1082, University Hospital Milétrie, Poitiers, 86000 France; Center of Clinical Investigation, Inserm 0802, Poitiers, 86000 France; Department of Anesthesiology, Inserm U 1116, University Hospital of Strasbourg, Nancy, 54000 France; Intensive Care Unit, University Hospital Pontchaillou, Rennes, 35000 France; Intensive Care Unit, University Hospital Côte de Nacre, Caen, 14000 France; Intensive Care Unit, University Hospital Henri Mondor, Créteil, 94010 France; Critical Care Department, University Hospital of Angers, Angers, 49100 France; Intensive Care Unit, University Hospital of Angers, Angers, 49100 France; Intensive Care Unit, University Hospital of Rouen, Rouen, 76000 France; Intensive Care Unit, Hospital Les Oudairies, La Roche-sur-Yon, 85925 France; Department of Anesthesiology, University Hospital of Clermont-Ferrand, Clermont-Ferrand, 63000 France; Critical Care Department, University Hospital Bretonneau, Tours, 37000 France; Intensive Care Unit, University Hospital of Limoges, Limoges, 87042 France; Center of Clinical Investigation, INSERM 1435, CHU Dupuytren, 2 Avenue Martin Luther King, Limoges, 87042 France

## Abstract

**Introduction:**

Circulatory failure during brain death organ donor resuscitation is a problem that compromises recovery of organs. Combined administration of steroid, thyroxine and vasopressin has been proposed to optimize the management of brain deceased donors before recovery of organs. However the single administration of hydrocortisone has not been rigorously evaluated in any trial.

**Methods:**

In this prospective multicenter cluster study, 259 subjects were included. Administration of low-dose steroids composed the steroid group (n = 102).

**Results:**

Although there were more patients in the steroid group who received norepinephrine before brain death (80% vs. 66%: *P* = 0.03), mean dose of vasopressor administered after brain death was significantly lower than in the control group (1.18 ± 0.92 mg/H vs. 1.49 ± 1.29 mg/H: *P* = 0.03), duration of vasopressor support use was shorter (874 min vs. 1160 min: *P* < 0.0001) and norepinephrine weaning before aortic clamping was more frequent (33.8% vs. 9.5%: *P* < 0.0001). Using a survival approach, probability of norepinephrine weaning was significantly different between the two groups (*P* < 0.0001) with a probability of weaning 4.67 times higher in the steroid group than in the control group (95% CI: 2.30 – 9.49).

**Conclusions:**

Despite no observed benefits of the steroid administration on primary function recovery of transplanted grafts, administration of glucocorticoids should be a part of the resuscitation management of deceased donors with hemodynamic instability.

## Introduction

Currently in France, increasing transplantation indications cannot be met because of graft shortage. It has been proven that the amount of procured grafts can be increased by an optimized management of brain-dead patients [1–5]. Almost 80% of brain-dead patients exhibit circulatory failure and it is commonly associated with heart, lung, kidney, liver and pancreas dysfunction, which compromises organ procurement in 10% to 25% of cases [6]. Although circulatory failure is controlled in more than 60% of brain-dead patients, primary function recovery of the grafts, especially heart, liver and pancreas grafts can be altered by increased vascular filling and administered vasopressors [7–9].

Thyroid hormones and cortisol deficit have already been identified as circulatory failure factors [10–12]. Thus, current British guidelines recommend giving thyroid hormones and corticosteroids to brain-dead patients with circulatory failure [13–15]. Several studies showed an increased amount of procured grafts and less primary dysfunction in transplanted heart grafts when triple therapy with thyroid hormone, corticosteroids and arginine vasopressin was used [6, 16]. However, systematic triple therapy remains debated because these studies are retrospective, whereas donor characteristics have considerably evolved with time (mean age of included patients in these studies is significantly inferior to the mean age of present potential donors for example), because the groups are not comparable and because the respective contribution of each agent of the triple therapy administered remains controversial.

This prospective multicenter study aimed to demonstrate that systemic administration of low-dose steroids during resuscitation of brain-dead donors makes vasopressor weaning possible in 25% of patients and also decreases by more than 15% the quantity of vasopressors needed to control circulatory failure. It also aimed at studying the impact of steroid administration on primary function recovery of grafts and on the number of procured grafts compared to the amount of potential donors who died with brain death.

## Material and methods

The study was approved by the French human subject protection committee (CPP OUEST III, number 061026), which waived the need for written informed consent from the family. In fact, French law entitles the conduct of randomized studies among clinically deceased patients without any informed consent from the family of the patient concerned. The study was conducted in accordance with the ethical standards of the Declaration of Helsinski as well as the Declaration of Istanbul and in compliance with French guidelines on organ procurement. The families of the donors were informed of the study. The French *Registre National des Refus* was consulted systematically (mandatory in France) to eliminate any opposition of the donor to participation in a clinical trial or organ donation. The authors decided to conduct a prospective cluster study involving 22 ICUs during 15 months to compare two different resuscitation strategies: systematic hydrocortisone supplementation (steroid group) or no supplementation (control group) in brain-dead patients who were potential organ donors. Among the involved centers, 11 gave standard-care, low-dose hydrocortisone to brain-dead patients before organ procurement and 11 did not use that therapy. All the patients were treated in the same way in a given center and all the centers introduced vasopressor support on the same hemodynamic criteria so minimizing the bias bound to the absence of randomization and to the potential center effect.

### Patients

All brain-dead patients, over 18 years old, hospitalized in the involved centers and considered for organ procurement were eligible for the study. Brain death was determined according to the usual criteria of French law: no motor response to nociceptive stimuli, no more brain stem reflexes and no more spontaneous breathing, which was confirmed with an apnea test when there were no other confounding factors such as hypothermia, collapsus and impregnation of the central nervous system by depressant drugs. Patients were included once the family has been informed about the study protocol. Patients over 18 years old, who received corticosteroids or had adrenal insufficiency before brain death, were excluded. Patients registered in *Registre National des Refus* (in France, where refusals to donate organs are registered) were secondarily excluded from the study. Two groups of equal numbers and patient ages could not be obtained because of the illegality of randomization of brain-dead patients in France and because of the cluster distribution of recruited patients.

### Hemodynamic evaluation

All patients were monitored through an artery catheter and a central venous catheter in the superior vena cava. Patients received controlled ventilation with a tidal volume (VT) of 7 to 10 ml/kg, positive end-expiratory pressure (PEEP) of 0 to 5 cmH_2_O, and an adjusted rate to maintain normocapnia. Hypotension was defined as mean blood pressure <65 mmHg. Mean blood pressure was set between 65 and 75 mmHg. The preload dependence was evaluated with respiratory variation of pulse blood pressure or with variation of subaortic doppler speeds or echocardiography-measured vena cava diameter; common threshold values previously defined in the protocol were used [17]. Vascular filling was performed with 500- to 1,000-ml aliquots of crystalloids administered during 10 to 15 minutes. Norepinephrine therapy was started if persistent hypotension and no preload dependence criteria were noted. If the mean blood pressure was superior to 85 mmHg, norepinephrine dose was decreased until complete vasopressor weaning.

When diabetes insipidus (defined by diuresis >3 ml/kg/h and urine density <1003 gm/cm^3^) occurred, diuresis needed to be offset volume for volume and desmopressin was administered to maintain diuresis between 1 and 3 ml/kg/h and natremia <160 mmol/L.

### Study design

Administration of replacement dose of hydrocortisone had to be started at a maximum 6 h after the diagnosis of brain death. Adrenal stimulation by adrenocorticotrophic hormone (ACTH) (250 μg injection of tetracosactrin, Synacthen^R^; Novartis Pharma SAS, Rueil Malmaison, France) was investigated. Adrenal insufficiency was defined by plasma cortisol level inferior to 18 μg/dl at time of injection (zero minutes, T0) and/or by a variation of plasma cortisol level following ACTH injection (T60 to T0) inferior to 9 μg/dl (so-called non-responding patients) [18]. After ACTH injection, when plasma cortisol level was superior to 9 μg/dl, patients were classed as responding. Patient then received a 50-mg injection of hydrocortisone (Roussel-Uclaf, Romainville, France) followed by a continuous infusion of 10 mg/h until the aortic clamping was performed in the operating room during organ retrieval. Plasma cortisol assays were done before ACTH injection (T0) and 60 minutes after injection (T60) by electrogenerated chemiluminescence (Roche automat modular). In the control group (patients did not receive hydrocortisone), the physician in charge of the patient decided whether or not to perform the ACTH test.

### Appraisal criteria

The main appraisal criterion was the quantity of norepinephrine weaning possible during resuscitation of brain-dead donors, or the decrease of the quantity of vasopressors needed to control circulatory failure. It was quantified with the average dose per hour, the variation percentage and the duration of administration of norepinephrine from the time of inclusion into the study (when brain death was diagnosed) to the aortic clamping during organ procurement.

Secondary appraisal criteria were: number of recovered organs compared with number of brain-dead donors and with number of organs considered for procurement when grafts were proposed to the *Agence de la biomédecine* (French organization responsible for census of brain-dead patients and for national distribution of grafts). Another secondary criterion was the frequency of delayed graft function (DGF) for each graft. For each organ, DGF was determined from clinical and biological data usually considered by French transplant physicians (Table 1). Pancreas grafts were counted among recovered organs but primary dysfunction analyses were not studied since these organs were recovered for islets of Langerhans transplantation and not for whole organ transplantation. Finally, cold ischemia duration was registered for each recovered and transplanted organ.

**Table 1:** **Delayed graft function (DGF) criteria**

Organs	DGF criteria
Kidney	Hemodialysis during first week or creatinine >250 μmol/L at day + 10
Liver	ASAT >1,500 or ALAT >1,000 or Quick Time <30% at h + 72
Heart	Circulatory support or left ventricle dysfunction or right ventricle dysfunction with pulmonary hypertension
Lungs	PaO2/FiO2 > 100 mmHg and <300 mmHg and/or pulmonary edema

PaO_2_/FiO_2_, arterial partial pressure of oxygen/inspired oxygen fraction.

ASAT: Aspartate Aminotransferase.

ALAT: Alanine Aminotransferase.

### Statistical analysis

Statistical analyses were performed using the SAS 9.2 software package (SAS Inc, Cary, NC, USA) and Statview 5.0 software (SAS Institute, Berkeley, CA, USA). Continuous variables were expressed as mean ± SD and qualitative variables were expressed as absolute numbers and percentages. Comparisons between the steroid group and control group were performed using the Student *t*-test, or Mann-Whitney *U*-test when appropriate for the quantitative variables, and the chi-square test for the qualitative variables. For paired donor-kidneys, comparison of DGF between the two groups was adjusted for cold ischemia duration, serum creatinine value, norepinephrine dose, and simplified acute physiological score II (SAPSII), using logistic regression analysis with random effects to account for impairment, performed using the PROC GLIMMIX command in SAS. Kaplan-Meier curves were plotted to describe the probability of norepinephrine weaning. Curves were compared between groups using the log-rank test. The corresponding hazard ratio (HR) was estimated using a univariate Cox model. All statistical tests were two-sided and were conducted using the 0.05 level of significance.

## Results

**Figure 1:**
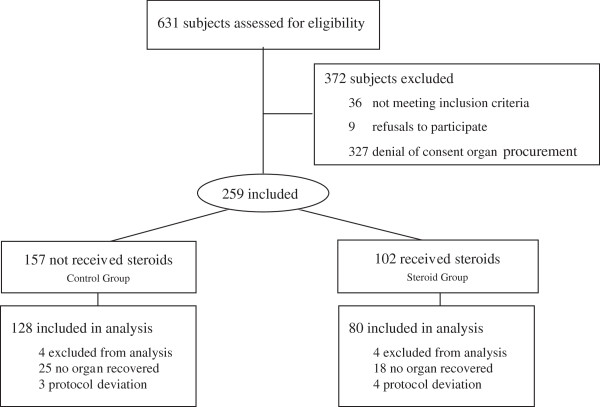
**Study flow chart.**

During the study, 631 brain-dead patients were hospitalized in the 22 participating centers. Hemorrhage within the brain or meninges represented 72% of the etiologies of brain insult responsible for brain death (Table 2). Organs were recovered from 304 patients (48%) and 259 donors (41%) were included in the study. Finally, 208 donors (33%) were analyzed: 128 brain-dead patients were included in the control group and 80 in the steroid group (Figure 1). The mean age of each group was similar but in the steroid group, the number of patients over 65 years old was significantly higher, with an initial severity score represented by the highest SAPSII (Table 2). The mean dose of hydrocortisone received in the steroid group was 210 ± 35 mg.

**Table 2:** **Global population - general characteristics**

	All (n = 208)	Control group (n = 128)	Steroid group (n = 80)	***P***-value
**Characteristics**				
Age, years, mean (SD)	51.1 (16.7)	48.1 (16.1)	56.1 (16.6)	0.77
Age >65 years, n (%)	44 (21.1)	17 (13.3)	25 (31.2)	0.001
Sex ratio	1.39	1.42	1.35	0.87
Body mass index, mean (SD)	25.3 (4.7)	25.5 (4.7)	25.1 (4.8)	0.54
Simplified acute physiology score, h 24, mean (SD)	52.8 (19.4)	47.6 (19.8)	58.9 (17.1)	0.0001
Cortisol time 0 minutes, μg/dl, mean (SD)	17.8 (14.2)	20.2 (14.1)	16.5 (14.2)	0.16
Cortisol time 60 to 0 minutes, μg/dl, mean (SD)	16.9 (16.8)	16.2 (19.6)	18.7 (19.1)	0.48
Adrenal insufficiency, n (%)	94/121 (77.6)	30/41 (73)	64/80 (80)	0.39
Adrenocorticotrophic hormone responders, n (%)	77/121 (63.6)	26/41 (63.4)	51/80 (63.7)	0.97
Average length of support, h, mean (SD)	21 (8.5)	21.5 (8.15)	19.4 (9.5)	0.007
**Brain death etiology**				
Traumatic brain injury, n (%)	60 (28.8)	35 (27.3)	25 (31.2)	0.54
Brain hemorrhage, n (%)	83 (39.9)	57 (44.5)	26 (32.5)	0.08
Subarachnoid hemorrhage, n (%)	67 (32.2)	48 (37.5)	19 (23.7)	0.03
Cerebral ischemic injury, n (%)	21 (10.1)	12 (9.4)	9 (11.2)	0.66
Anoxic encephalopathy, n (%)	31 (14.9)	19 (14.8)	12 (15)	0.97
Post neurosurgery, n (%)	4 (1.9)	4 (3.1)	0	0.11

**Figure 2:**
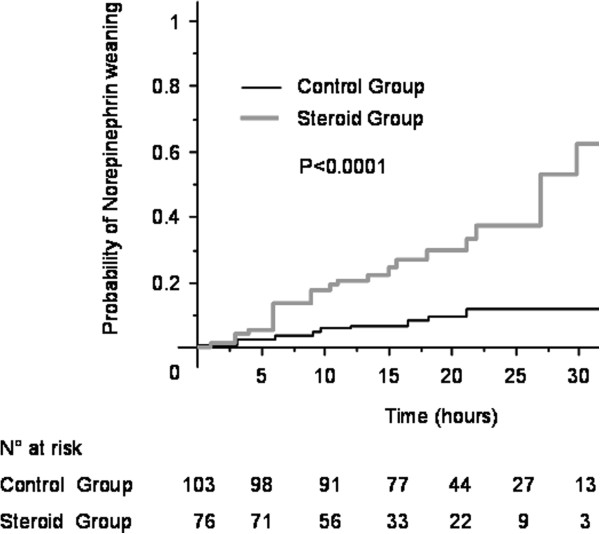
**Kaplan-Meier curves for the probability of continuation of norepinephrine.**

The ACTH stimulation test was performed in the 80 patients in the steroid group and in 41 patients in the control group; it revealed adrenal insufficiency in 94/121 brain-dead patients (78%). In the steroid group, the mean time before administration of hydrocortisone was 168 ± 130 minutes after brain death diagnosis. The mean quantity of hypotension episodes was comparable in control group and steroid group (1.2 ± 1.4 versus 1.0 ± 1.6, *P* = 0.18). The mean vascular filling volume per hour was similar in the two groups (179 ± 106 ml/h versus 219 ± 165 ml/h, *P* = 0.88). Although there were more patients in the steroid group who received norepinephrine before brain death (80% versus 66%, *P* = 0.03), the mean dose of vasopressor administered after brain death was significantly lower than in the control group (1.18 ± 0.92 mg/h versus 1.49 ± 1.29 mg/h, *P* = 0.03), duration of vasopressor support use was shorter than in control group (874 minutes versus 1,160 minutes: *P* <0.0001) and norepinephrine weaning before aortic clamping was more frequent (33.8% versus 9.5%, *P* <0.0001) (Table 3). Using a survival approach, probability of norepinephrine weaning was significantly different between the two groups (*P* <0.0001) with a probability of weaning 4.67 times higher in the steroid group than in the control group (95% CI 2.30, 9.49) (Figure 2). For the sub-groups of patients responding or non-responding to the ACTH test, no significant differences in norepinephrine weaning were noted (HR 1.84, 95% CI 0.67, 5.05, *P* = 0.23 and HR 6.74, 95% CI 0.82, 55.19, *P* = 0.07, respectively).

**Table 3:** **Hemodynamic results**

	Control group (n = 128)	Steroid group (n = 80)	***P***-value
Patients with norepinephrine before inclusion, n (%)	85 (66.4)	64 (80)	0.03
Patients with norepinephrine during support, n (%)	103 (80.4)	76 (95)	0.12
Norepinephrine dose (mg/h), mean (SD)	1.49 (1.29)	1.18 (0.92)	0.03
Patients weaned norepinephrine, n (%)	10/103 (9.7)	26/76 (34.2)	<0.0001
Varying dose in unweaned patients, % of initial dosing	+ 46	-20.9	0.0004
Duration with norepinephrine, minutes, median	1160	874	<0.0001

Varying dose in unweaned patients = (norepinephrin end dosing – norepinephrin initial dosing)/norepinephrin initial dosing.

The number of recovered organs compared to the number of brain-dead patients was similar in the steroid group and control group (3.31 ± 1.36 versus 3.51 ± 1.39, *P* = 0.23) (Table 4). However, if compared to the number of organs considered for procurement, the percentage of recovered grafts in the steroid group was higher than in control group, but it did not reach the significance threshold (92% versus 88%, *P* = 0.07) (Table 4). There was no significant difference in cold ischemia duration between the two groups (Table 5).

**Table 4:** **Organs recovered/organs recoverable (%)**

Organs	All patients (n = 208)	Control group (n = 128)	Steroid group (n = 80)	***P***-value
Kidney	394/403 (97.7)	243/248 (98)	151/155 (97.4)	0.65
Liver	162/172 (94.2)	99/105 (94.3)	63/67 (94)	0.61
Heart	66/80 (82.5)	47/56 (83.9)	19/24 (79.1)	0.74
Lung	71/93 (73.9)	44/62 (70.9)	27/34 (79.4)	0.36
Pancreas	21/47 (44.6)	16/39 (41)	5/8 (62.5)	0.43
Total	714/798 (89.5)	449/510 (88)	265/288 (92)	0.07
Organs/donors, n (SD)	3.43 (1.37)	3.51 (1.39)	3.31 (1.36)	0.23

Results presented as number/total number (%) unless stated otherwise.

**Table 5:** **Cold ischemia duration (hours) by organ and strategy, mean (SD)**

Organs	Control group	Steroid group	***P***-value
Right kidney	19.05 (6.84)	19.07 (6.4)	0.88
Left kidney	16.08 (5.76)	16.12 (4.23)	0.51
Liver	8.51 (2.8)	8.76 (2.2)	0.31
Lung	5.85 (1.65)	5.21 (1.48)	0.25
Heart	3.04 (1.1)	3.39 (0.97)	0.24
Pancreas	10.94 (1.47)	11.68 (1.15)	0.55

Among the 714 recovered grafts, 72 grafts were not transplanted (10.1%). Among the 642 transplanted and studied grafts, we observed a DGF in one case out of three (212/642). DGF of kidney graft was more frequent in the steroid group (39% versus 28%, *P* = 0.03) (Table 6). However this difference did not persist after adjustment for the other variables: logistic regression with random effects for donor showed that the probability of kidney graft DGF increased with age of the donor (*P* = 0.0007), and with serum creatinine value before procurement (*P* = 0.006) and decreased with norepinephrine dose (*P* = 0.03) but was not modified by the strategy (*P* = 0.26). When SAPSII was considered in the model instead of age, the only predictors of kidney graft DGF were serum creatinine value (*P* = 0.04) and cold ischemia duration (*P* = 0.01).

**Table 6:** **Delayed grafts function (DGF) by organ and strategy, number (%)**

Organs	Control group	Steroid group	***P***-value	Adjusted ***P***-value*
Kidney	65/230 (28.3)	55/141 (39)	0.03	0.04
Liver	25/94 (26.6)	22/59 (37.3)	0.16	0.11
Lung	12/31 (38.7)	5/25 (20)	0.13	0.21
Heart	21/44 (47.7)	7/18 (38.8)	0.52	0.45
Total	123/399 (30.8)	89/243 (36.6)	0.14	

*Adjusted for cold ischemia duration.

For the other grafts, DGF frequency was comparable in both groups (Table 6).

## Discussion

This multicenter prospective controlled study demonstrates that systemic administration of low-dose steroids during brain-death resuscitation of potential brain-dead organ donors makes vasopressor weaning possible in more than a third of patients (33.8%) and also decreases by more than 20% the quantity of vasopressors needed to control circulatory failure, allowing for a significant reduction in the need for inotropic support. This effect is not related to adrenal insufficiency identified by ACTH stimulation, but steroid administration alone fails to increase the number of organ recovered for transplantation.

When an organ donor with brain-death is resuscitated, one of the main objectives is to stabilize the hemodynamic state in order to limit ischemia and inflammation as far as possible in the different organs. This goal is usually achieved by a combination of fluid expansion and inotrope administration. Norepinephrine is associated with a decreased rate of high-yield procurement and it seems clinically relevant to reduce doses or use of norepinephrine in brain-dead donors to increase the rate of organ procurement. Thyroid hormones and cortisol deficit have already been identified as circulatory failure factors in brain-dead donors [10–12]. Our study confirms that prescription of a replacement dose of hydrocortisone during resuscitation of a potential brain-dead donor makes vasopressor weaning possible and also decreases vasopressor doses, which are necessary to maintain a stable hemodynamic state in unweaned patients. These results are in agreement with those of a single-center observational cohort of 30 patients with brain death who were administered 50 mg of hydrocortisone: in 58% of patients norepinephrine doses were reduced by 30% after three hours [19]. Another study compared two groups of donors including during two consecutive periods and studied the impact of high doses of methylprednisolone (15 mg/kg) versus low doses of hydrocortisone (300 to 500 mg): frequency of vasopressor weaning was 39% in the first group and 47% in the second group, with no significant difference between both groups [20]. Our results for hemodynamic stability and decrease in vasopressor use following steroid administration are similar to the results obtained in several studies, in which identical doses of hydrocortisone were administered in patients who were not brain-dead [21, 22].

Although in our study, adrenal insufficiency frequency (77.6%) was similar to the results of both studies cited above [19, 20], no significant difference was noted in the frequency of vasopressor weaning of brain-dead patients compared to plasma cortisol level at baseline or to initial response to ACTH stimulation. Our results did not confirm those of Nicolas-Robin *et al*. [19], who suggested that steroid administration would be more beneficial in patients with documented adrenal insufficiency. However our results are consistent with other studies conducted in different settings, such as in septic shock, showing that the ACTH stimulation test had no predictive value for hemodynamic response of patients receiving corticosteroids [23–26].

Several reports suggest that multimodal hormonal therapy (thyroid hormone, corticosteroids and arginine vasopressin) might have beneficial effects on recovery of organs, with an increased amount of procured grafts and less primary dysfunction in transplanted heart grafts [6, 16]; however, the respective contribution of each hormonal therapy remains controversial. Thyroid hormone administration by itself has been considered either to be beneficial, neutral or to have no significant impact on organ procurement. In our study, no significant difference was noted in the number of recovered organs per donor in either group. The number of recovered organs compared to the number of organs considered for procurement was slightly higher in the steroid group than in the control group, but the difference was not significant. DGF frequency was similar in both groups regardless of the grafted organ. Kidney grafts were the only exception with primary dysfunction significantly more frequent in the steroid group. Despite the benefits of a replacement dose of hydrocortisone on vasopressor consumption, our study did not demonstrate any benefits of steroid administration for primary function recovery of transplanted grafts. Some studies have either shown the absence of clinical impact of a replacement dose of hydrocortisone on primary function recovery of kidney [27], liver [28], heart and lung grafts; for those grafts, only a decrease in systemic inflammatory markers and their expression in tissue, and a decrease of extravascular lung water accumulation were beneficial for primary graft recovery [29, 30].

Our study has several limitations: lack of randomization of brain-dead patients, cluster distribution of recruited patients, lack of data about receivers’ history, and comorbidities of receivers and their consequences for DGF. This might have limited our capacity to identify some potential beneficial effects of steroid administration during brain-dead donor resuscitation and we expect that practitioners would not change their usual practice in brain-dead donor resuscitation.

## Conclusion

Early substitutive administration of glucocorticoids in a potential brain-dead organ donor with circulatory failure makes it possible to significantly reduce the cumulative dose and administration duration of vasopressors. Whatever the case, based on our results we cannot reach a conclusion as to whether or not the routine use of steroids administration in potential brain-dead organ donors should be supported, independently of documented relative adrenal insufficiency, and no benefits for primary function recovery of transplanted grafts were observed in the study. Existing controversy in the literature suggests that a multiple strategy is required to achieve measurable effects in the standard care of organ donors. Routine steroid administration is probably an important component of that strategy to improve recovery of organs following brain death, but should not be used alone and probably should be considered along with other hormonal factors, for which the respective contribution remains to be defined.

## Key messages

-  Steroids and norepinephrine are equally effective in achieving hemodynamic stability in various different groups of brain-dead organ donors.
-  Steroid administration alone fails to increase the number of organs recovered for transplantation.
-  Steroid administration did not demonstrate any benefits for primary function recovery of transplanted grafts.
-  The decision to use steroids or norepinephrine was not observed to affect meaningful outcomes for hemodynamically stable brain-dead organ donors.

## Abbreviations

ACTH: adrenocorticotrophic hormone
CH: Hospital Center
CHU: University Hospital Center
CPP: *Comité de Protection des Personnes*
DGF: delayed graft function
HH: hemisuccinate hydrocortisone
HR: hazard ratio
PaO_2_/FiO_2_: arterial partial pressure of oxygen/inspired oxygen fraction
PEEP: positive end-expiratory pressure
SAPS: simplified acute physiological score
T0: time of injection
T60: 60 minutes after injection
VT: tidal volume.
